# The Impact of the COVID-19 Pandemic on Orthopaedic Knee Procedures: A National Multicentered Analysis

**DOI:** 10.7759/cureus.31681

**Published:** 2022-11-19

**Authors:** Jordan Pizzarro, Haig Pakhchanian, Omar Tarawneh, Ivan Liu, Rahul Raiker, Jordan S Cohen, Alisa Malyavko, Sean Tabaie, Savya Thakkar

**Affiliations:** 1 Orthopaedic Surgery, George Washington University School of Medicine and Health Sciences, Washington, D.C., USA; 2 Ophthalmology, George Washington University School of Medicine and Health Sciences, Washington, D.C., USA; 3 School of Medicine, New York Medical College, Valhalla, USA; 4 Ophthalmology, Medical College of Georgia, Augusta, USA; 5 Ophthalmology, West Virginia University School of Medicine, Morgantown, USA; 6 Orthopaedic Surgery, Perelman School of Medicine at the University of Pennsylvania, Philadelphia, USA; 7 Orthopaedic Surgery, Children's National Hospital, Washington, D.C., USA; 8 Orthopaedic Surgery, Johns Hopkins Health System, Baltimore, USA

**Keywords:** coronavirus disease 2019, current trends, orthopaedics surgery, knee arthroplasty, knee procedure, covid-19

## Abstract

Introduction: The coronavirus disease 2019 (COVID-19) pandemic strained the United States healthcare system, and associated policies resulted in the postponement or cancellation of many elective surgeries. While most orthopaedic surgeons are aware of how the pandemic affected their patients’ care, broader national trends in the operative treatment of orthopaedic knee pathology are poorly characterized. Therefore, the purpose of this study was to identify trends in orthopaedic knee procedures during the COVID-19 pandemic.

Methods: The TriNetX database was queried for orthopaedic knee procedures performed from March 2018 to May 2021. Procedures were classified as arthroplasty (total knee arthroplasty (TKA), revision total knee arthroplasty) or non-arthroplasty (tendon or ligament repair, fracture fixation). Procedural volume per healthcare organization was determined over five seasons from March 2020 to May 2021 and compared to overlapping pre-pandemic periods from March 2018 to May 2019. Descriptive analysis was performed, and comparisons were made using a Student’s T-test.

Results: Compared to the pre-pandemic period, there were significant decreases in primary TKA (p=0.016), femoral or entire tibial component revision TKA (p=0.005), and open treatment of femoral shaft fractures (p=0.007) in spring 2020. Procedural volume returned to baseline in summer 2020 through winter 2021. In spring 2021, primary TKA (p=0.017) and one component revision TKA (p=0.003) increased compared to the pre-pandemic period.

Conclusion: The greatest decrease in knee procedures occurred early in the pandemic. Rates of these procedures have since rebounded, with some exceeding pre-pandemic levels. Hospitals are now better able to accommodate orthopaedic surgical volume while continuing to care for patients with COVID-19.

## Introduction

First discovered in China in December 2019, coronavirus disease 2019 (COVID-19) quickly spread and became a major public health emergency. On March 11, 2020, the World Health Organization declared COVID-19 a global pandemic [1]. As of July 2021, over 33.5 million cases had been reported with over 600,000 deaths in the United States alone [2]. COVID-19 put great demands on the healthcare system as the number of critically ill patients within hospitals increased. Many techniques, such as social distancing and wearing of masks, were implemented throughout the country to decrease the spread of COVID-19. The American College of Surgeons (ACS) released statements regarding changes in practice to minimize the viral spread and preserve resources, including minimizing the use of ICU beds, personal protective equipment, and ventilators [3]. However, one of the biggest changes was the recommendation to cancel or postpone all elective surgeries until the healthcare infrastructure could support them.

Due to the elective nature of most orthopaedic surgery, our field was particularly vulnerable to these restrictions [4,5]. According to a 2019 Blue Cross Blue Shield report, elective orthopaedic procedures alone accounted for approximately 47% of total orthopaedic spending [5]. Elective musculoskeletal surgery generates $15.6-$21.1 billion in net income per year for hospitals in the United States [6]. To help determine which procedures should be prioritized, the American Academy of Orthopedic Surgeons (AAOS) released a tiered system to classify surgeries: elective (delaying surgery will not cause harm to the patient), urgent elective (some outpatient surgeries could be performed depending on the availability of resources), urgent (injuries for which immediate treatment could prevent impaired function), and emergent (life- and limb-threatening injuries) [7].

Knee operations are extremely common, and the number of knee surgeries including elective knee procedures has continued to increase [4,5]. In 2011, knee arthroplasty alone accounted for 4.6% of all operating room procedures, making it the third most common procedure in the United States [8]. Elective knee arthroplasties increased by 17% from 2010 to 2017 [5]. Other knee procedures have also become increasingly prevalent [9,10].

It has been estimated that the first eight weeks of elective orthopaedic surgical cancellation during the pandemic led to losses of $2.6-$3.5 billion in net income for United States hospitals [6]. Given the prevalence of knee joint procedures, it is important to understand how they have been impacted by pandemic restrictions. Therefore, the purpose of this study was to identify trends in orthopaedic knee joint procedures during the COVID-19 pandemic.

## Materials and methods

A retrospective study was performed using data from March 2018 to May 2021 obtained from the TriNetX Research Network database (Cambridge, Massachusetts, United States). TriNetX is a global federated database consisting of electronic medical records from 51 healthcare organizations and over 68 million patient records. The TriNetX platform consists of real-time, de-identified, aggregate patient data including demographics, vitals, genomics, medications, and procedures [11]. The data on the TriNetX platform is considered Health Insurance Portability and Accountability Act (HIPAA) compliant as protected health information is not provided. Most participating institutions provide data dating back to the last seven years. TriNetX has been previously used in other orthopaedic studies [12,13].

Using Current Procedural Terminology (CPT) codes (Table 1), TriNetX was queried for common orthopaedic knee procedures. This included both inpatient and outpatient visits. Common procedures were then stratified into arthroplasty and non-arthroplasty groups. Arthroplasty included CPT codes for total knee arthroplasty (TKA) and TKA revisions. The non-arthroplasty group included tendon and ligament procedures, quadriceps and hamstring rupture repair, patella dislocation reconstruction, and fractures. The fracture cohort was further stratified into femoral shaft, patella, and tibial plateau. The coded procedures are more fully described in Table 1. The data was then analyzed based on seasonal averages and compared to levels before the pandemic (2018-2019). The seasons analyzed included spring 2020 (March-May 2020), summer 2020 (June-August 2020), fall 2020 (September-November 2020), winter 2020/2021 (December 2020-February 2021), spring 2021 (March-May 2021), and the pandemic period overall (March 2020-May 2021) compared to overlapping months between March 2018 and May 2019. To normalize data provided by TriNetX, the reported figures are the average number of encounters per healthcare organization (HCO). The average monthly encounters during each season during the pandemic were compared with the average monthly encounters from overlapping months in 2018 and 2019. Descriptive analyses were performed, and comparisons were made using a student’s t-test. Statistical analysis was performed using Microsoft Excel (Microsoft Corporation, Redmond, Washington, USA).

**Table 1:** CPT Codes for Analyzed Knee Procedures CPT: Current Procedural Terminology

Procedure	CPT Code
Arthroplasty	
Arthroplasty, knee, condyle and plateau; medial AND lateral compartments with or without patella resurfacing (total knee arthroplasty)	27447
Revision of total knee arthroplasty, with or without allograft; 1 component	27486
Revision of total knee arthroplasty, with or without allograft; femoral and entire tibial component	27487
Non-Arthroplasty	
Suture of infrapatellar tendon; primary	27380
Suture of quadriceps or hamstring muscle rupture; primary	27385
Repair, primary, torn ligament and/or capsule, knee; collateral	27405 OR 27407 OR 27409
Reconstruction of dislocating patella	27420 OR 27422
(1) Open treatment of femoral shaft fracture, with or without external fixation, with insertion of intramedullary implant, with or without cerclage and/or locking screws (2) Open treatment of femoral shaft fracture with plate/screws, with or without cerclage	27506 OR 27507
Open treatment of patellar fracture, with internal fixation and/or partial or complete patellectomy and soft tissue repair	27524
(1) Closed treatment of tibial fracture, proximal (plateau); without manipulation (2) Closed treatment of tibial fracture, proximal (plateau); with or without manipulation, with skeletal traction	27530 OR 27532
Open treatment of tibial fracture, proximal (plateau)	27535 OR 27536

## Results

Compared to the pre-pandemic period, the beginning of the pandemic in spring (March to May) 2020 saw significant decreases in primary TKA (24.9 vs 44.2, p=0.016), femoral or entire tibial component revision TKA (4 vs 5.6, p=0.005), and open treatment of femoral shaft fractures (5.4 vs 6.8, p=0.007). Summer 2020 through winter 2021 saw no significant differences in any of the analyzed knee procedures compared to the pre-pandemic period (p>0.05). Spring 2021 saw significant increases in primary TKA (55 vs 44.2, p=0.017) and one component revision TKA (3.8 vs 3, p=0.003) compared to the pre-pandemic period. Comparing the entire pandemic to the pre-pandemic period, the only significant decreases seen were for femoral or entire tibial component revision TKA (4.7 vs 5.3, p=0.015) and reconstruction of dislocating patella (2 vs 2.3, p=0.041).

**Figure 1:**
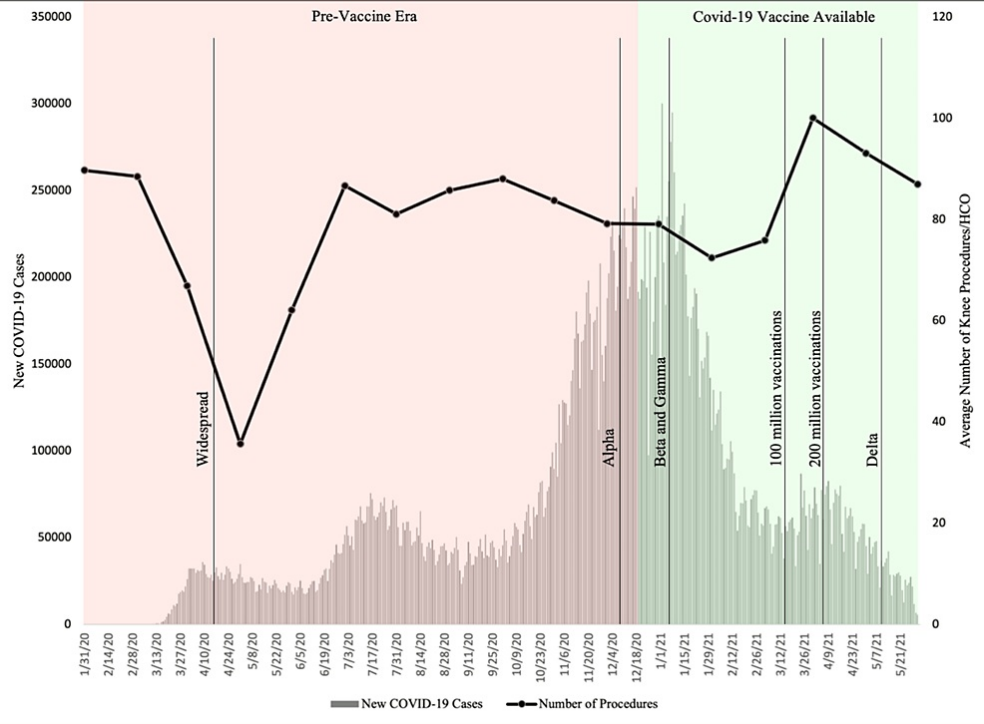
Knee Procedural Trends During COVID-19 Pandemic HCO: Healthcare Organization; COVID-19: Coronavirus Disease 2019

Figure 1 shows the monthly total number of knee procedures per healthcare organization (HCO) over time overlaid onto a temporal bar graph of new COVID-19 cases, giving an indication of the impact each wave of the pandemic had on orthopaedic knee procedures. The spread of various COVID-19 variants as well as vaccination availability in the United States are indicated on the graph. The largest reduction in the number of procedures was seen soon after COVID-19 was declared a global pandemic in the spring of 2020, but this was followed by a large rebound in early summer of 2020. Although COVID-19 became widespread in the spring of 2020, the greatest surge in cases was not seen until November 2020 to February 2021 when the alpha, beta, and gamma variants were making their way through the country. Although not as drastic as the beginning of the pandemic, this surge was met with another steady decrease in knee procedures. By March 2021, 100 million vaccinations were given, rising to 200 million by the beginning of April. This was accompanied by a drastic decrease in COVID-19 cases as well as a large increase in knee procedures to levels higher than before the pandemic.

## Discussion

Given the high prevalence of orthopaedic knee procedures in the United States, it is important to understand how they have been affected during the COVID-19 pandemic. This study sought to characterize the trends in orthopaedic knee procedures performed during the COVID-19 pandemic period from March 2020 to May 2021. Overall, the initial seasons of the pandemic saw decreases in overall knee procedures while the later seasons saw increases compared to pre-pandemic means.

Several previous studies sought to characterize the initial effect of the COVID-19 pandemic on TKAs. Landy et al. examined the change in public interest in knee replacement and knee pain from December 2019 through May 2020 [14]. The authors reported that the number of online searches for knee replacements remained stable until early March 2020, but was followed by a large decrease, eventually plateauing at a daily search rate half of what it was prior to the pandemic at the end of the month. By the end of the search period in the middle of May, the search for knee replacements began increasing [14]. In line with these findings, another study using data within the Medicare population undergoing primary and revision TKA found a daily 94% decrease in late March 2020, leading to an 87% decrease in hospital Medicare revenue for arthroplasty [15].

Similarly, our study showed significant decreases in knee replacement procedures. The months of March 2020 to May 2020 were a period of tremendous challenges for the healthcare system as COVID-19 spread throughout the country. During this time, COVID-19 was declared a global pandemic and a national emergency in the United States. Travel bans and stay-at-home orders went into effect, all of which were compounded by a rising death toll. Additionally, initial recommendations for the cancellation of elective procedures were announced as COVID-19 patients increased in hospitals. Given the widespread fear of COVID-19 and the immense strain placed on the healthcare system, it is not surprising that this period was marked by significant decreases in some of the most common knee procedures.

Postponement of TKAs left patients continuing to manage the challenges that come with end-stage osteoarthritis and other degenerative diseases of the knee. A study conducted by Brown et al. characterized the impact that cancellation had on patients originally scheduled for TKA or THA in the spring and summer of 2020 [16]. In this study, 54% of patients reported that their pain increased since the cancellation, and 30% stated they would have proceeded with surgery despite the risks of COVID-19. Additionally, the highest causes of anxiety for these patients were uncertainty about when the procedure would be rescheduled and contracting COVID-19. However, 88% of patients reported that they intended to reschedule their surgery as soon as possible [16]. Our study found significant increases in primary and revision TKA in spring 2021 compared to pre-pandemic. Given the widespread availability of vaccines in early 2021, decreased COVID-19 cases, and the lifting of restrictions across the country, it is unsurprising that the healthcare system was able to accommodate the rebound in elective TKAs during these months.

While muscular, ligamentous, and tendon procedures as well as fractures showed overall decreases of 1-10% during the pandemic, few of these changes were significant. Most studies published in the literature analyzed the effects of COVID-19 on injuries and procedures during the initial months of the pandemic. A multicenter cohort study examined presentations of pediatric sports injuries, including meniscal and anterior cruciate ligament injury and patella dislocation, during the early (March to May 2020) and mid-pandemic (May to July 2020) period [17]. Within the first month of the pandemic, significant decreases in injuries were seen compared to pre-pandemic. However, when comparing early to mid-pandemic, no significant differences in injuries were found, and those occurring in the knee showed slightly increased incidence. Additionally, no delay in care was observed for these injuries that occurred during the pandemic. Compared to pre-pandemic, more injuries occurred at home due to a decrease in organized sports and most occurred while playing basketball and soccer, which are easily played at home [17]. Interestingly, the lack of sports participation during the pandemic may result in increased injury rates upon return to play, as suggested by a German elite soccer league that experienced a three-fold increase in injury rates following the COVID-19 lockdown [18]. Further research should be conducted to determine the effect of detraining on sports injury incidence following the resumption of organized sports.

Some studies have also examined the impact of the COVID-19 pandemic on fracture epidemiology. Despite an overall decrease of 19% for total fractures during the state-of-emergency period in 2020, Mitkovic et al. found no significant differences in the frequency of femoral or lower extremity fractures, except for the distal femoral fracture, compared to the same period in 2019 [19]. Increased falls by elderly individuals while on stay-at-home orders likely contributed to the increased incidence of distal femoral fractures during the analyzed pandemic period. In a level 1 pediatric trauma hospital, there was a nearly 60% decrease in fracture volume during March-April 2020, but no significant difference in the proportion of any lower extremity fracture [20]. Additionally, studies have shown that pediatric fractures had no delay in presentation and treatment during the first few months of the pandemic [17,20]. All of these findings support the results of our study.

This study has strengths that come with using the TriNetX database. This global database collects comprehensive patient data from over 130 healthcare organizations. Additionally, this is the first known study that has divided the pandemic period into seasons, allowing us to assess the impact of COVID-19 on knee procedures at various time points. However, this study is not without limitations. Despite being able to provide considerable amounts of patient information, data in the TriNetX database is only collected from participating organizations. Additionally, inconsistencies and inaccuracy of coding for diagnoses and procedures by hospitals and physicians may exist. Additionally, we were unable to stratify our results by geographic location; therefore, we are unable to report how the geographic variations in COVID-19 impacted orthopaedic cases.

## Conclusions

The initial months of the COVID-19 pandemic saw the greatest decreases in knee procedures, which have since rebounded with some reaching levels higher than they were before the pandemic. As orthopaedic knee procedures return to pre-pandemic levels while COVID-19 persists, it is important to consider how we can safely provide care for patients with orthopaedic pathology while caring adequately for those with COVID-19 and other medical problems.

## References

[1] Cucinotta D, Vanelli M (2020). WHO declares COVID-19 a pandemic. Acta Biomed.

[2] (2021). COVID Data Tracker Weekly Review. https://www.cdc.gov/coronavirus/2019-ncov/covid-data/covidview/index.html.

[3] (2021). COVID-19: Recommendations for Management of Elective Surgical Procedures. http://www.facs.org/covid-19/clinical-guidance/elective-surgery.

[4] Merrill C, Elixhauser A (2007). Hospital Stays Involving Musculoskeletal Procedures, 1997-2005. HCUP Statistical Brief #34. https://www.hcup-us.ahrq.gov/reports/statbriefs/sb34.pdf.

[5] (2021). Blue Cross Blue Shield Association: Planned knee and hip replacement surgeries are on the rise in the US. https://www.bcbs.com/the-health-of-america/reports/planned-knee-and-hip-replacement-surgeries-are-the-rise-the-us.

[6] Best MJ, Aziz KT, McFarland EG, Anderson GF, Srikumaran U (2020). Economic implications of decreased elective orthopaedic and musculoskeletal surgery volume during the coronavirus disease 2019 pandemic. Int Orthop.

[7] Guy DK, Bosco JA, Savoie III FH (2021). AAOS Guidelines for Elective Surgery During the Covid-19 Pandemic. American Academy of Orthopaedic Surgeons. https://www.aaos.org/about/covid-19-information-for-our-members/aaos-guidelines-for-elective-surgery/.

[8] Weiss AJ, Elixhauser A, Andrews RM (2014). Characteristics of Operating Room Procedures in U.S. Hospitals, 2011. HCUP Statistical Brief #170. https://www.hcup-us.ahrq.gov/reports/statbriefs/sb170-Operating-Room-Procedures-United-States-2011.jsp.

[9] Herzog MM, Marshall SW, Lund JL, Pate V, Mack CD, Spang JT (2018). Trends in Incidence of ACL Reconstruction and Concomitant Procedures Among Commercially Insured Individuals in the United States, 2002-2014. Sports Health.

[10] Abrams GD, Frank RM, Gupta AK, Harris JD, McCormick FM, Cole BJ (2013). Trends in meniscus repair and meniscectomy in the United States, 2005-2011. Am J Sports Med.

[11] (2022). TriNetX: Real-world data for the life sciences and healthcare. https://trinetx.com/.

[12] Kelly M, Turcotte J, Thomas D, Petre B, Morganti C, York J, Redziniak D (2021). Mid-term outcomes of anterior cruciate ligament reconstruction across age groups: a national database study. J Orthop.

[13] Menon N, Turcotte J, Patton C (2021). Structural allograft versus synthetic interbody cage for anterior cervical discectomy and fusion: a comparison of 1-year outcomes from a national database. Global Spine J.

[14] Landy DC, Chalmers BP, Utset-Ward TJ, Ast MP (2020). Public interest in knee replacement fell during the onset of the COVID-19 pandemic: a google trends analysis. HSS J.

[15] Barnes CL, Zhang X, Stronach BM, Haas DA (2021). The initial impact of COVID-19 on total hip and knee arthroplasty. J Arthroplasty.

[16] Brown TS, Bedard NA, Rojas EO (2020). The effect of the COVID-19 pandemic on electively scheduled hip and knee arthroplasty patients in the United States. J Arthroplasty.

[17] Johnson MA, Halloran K, Carpenter C (2021). Changes in pediatric sports injury presentation during the COVID-19 pandemic: a multicenter analysis. Orthop J Sports Med.

[18] Seshadri DR, Thom ML, Harlow ER, Drummond CK, Voos JE (2021). Case report: return to sport following the COVID-19 lockdown and its impact on injury rates in the German Soccer League. Front Sports Act Living.

[19] Mitkovic MM, Bumbasirevic M, Milenkovic S, Gajdobranski D, Bumbasirevic V, Mitkovic MB (2021). Influence of coronavirus disease 2019 pandemic state of emergency in orthopaedic fracture surgical treatment. Int Orthop.

[20] Bram JT, Johnson MA, Magee LC (2020). Where have all the fractures gone? The epidemiology of pediatric fractures during the COVID-19 pandemic. J Pediatr Orthop.

